# Whole-mol­ecule disorder of the heterometallic complex di­aqua-1κ^2^*O*-di­chlorido-2κ^2^*Cl*-(μ-2-formyl-6-meth­oxy­phenolato-1κ^2^*O*^1^,*O*^2^:2κ*O*^6^){μ-2-meth­oxy-6-[(methyl­imino)­meth­yl]phenolato-1κ^2^*N*,*O*^1^:2κ*O*^6^}lead(II)nickel(II)

**DOI:** 10.1107/S2056989025005857

**Published:** 2025-07-01

**Authors:** Olga Yu. Vassilyeva, Vladimir N. Kokozay, Evgeny Goreshnik

**Affiliations:** a Department of Chemistry, Taras Shevchenko National University of Kyiv, 12, Hetman Pavlo Skoropadskyi str., 01601 Kyiv, Ukraine; b Department of Inorganic Chemistry and Technology, Jozef Stefan Institute, Jamova 39, 1000 Ljubljana, Slovenia; Universidad de la Repüblica, Uruguay

**Keywords:** crystal structure, heterometallic complex, Schiff base ligand, *o*-vanillin, methyl­amine

## Abstract

The new mol­ecular complex [NiPb(C_9_H_10_NO_2_)(C_8_H_7_NO_3_)Cl_2_(H_2_O)_2_] with the Schiff base 2-meth­oxy-6-[(methyl­imino)­meth­yl]phenol and *ortho*-vanillin ligands, both deprotonated, crystallizes in the monoclinic space group *P*2_1_/*c* with one mol­ecule per asymmetric unit. The heterometallic mol­ecule exhibits a whole-mol­ecule orientational disorder [occupancy ratio 0.711 (6): 0.289 (6)] about a pseudo-twofold rotation axis that roughly bis­ects the mol­ecule along the Ni–Pb axis. The Ni centre coordination is distorted octa­hedral [Ni—N/O_eq_ = 1.996 (8)–2.021 (7) Å, major component] with two water mol­ecules in the apexes [2.088 (6) and 2.090 (5) Å]. The Pb atom is six-coordinate in a highly distorted tetra­gonal–bipyramidal geometry. Two phenolato and two methoxo O atoms from the deprotonated ligand moieties are nearly coplanar with the metal centre [Pb–O 2.301 (8)–2.740 (10) Å, major component], while the two chlorides are located on opposite sides of the plane [Pb—Cl = 2.821 (5) and 2.868 (5) Å]. The Ni–Pb pair of metals is bridged by two phenolato O atoms enabling a metal–metal separation of 3.441 (3)/3.477 (7) Å. Two intra­molecular O–H⋯Cl hydrogen bonds between coordinated aqua and chloride ligands appear to be a reason for the Cl–Pb–Cl and O–Ni–O angles being far from 180°. In the solid state, the well-separated heterometallic mol­ecules are inter­linked by π–π stacking and weak O—H⋯Cl and C—H⋯Cl/O hydrogen bonds. The mol­ecules show no significant inter­molecular inter­actions: the minimal *M*⋯*M* distance in the crystal is about 7.65 Å (Ni⋯Pb).

## Abstract 2

In the crystal the title compound exhibits full-mol­ecule disorder [occupancy ratio 0.711 (6): 0.289 (6)], generated by a false twofold rotation about the shorter, Ni–Pb, axis of the mol­ecule.

## 1. Chemical context

Heterometallic complexes comprising metals of different kinds are attractive objects of research in several important fields of scientific inter­est such as bioinorganic, medicinal and materials chemistry (Becker, 2024 ▸). Studying synthetic heterometallic compounds helps to understand the structure, bonding, and reaction mechanisms of natural metalloenzymes that feature multinuclear active sites, containing dissimilar metal ions. Ensued practical applications may lead to low-mol­ecular catalysts that are significantly more active, selective, or capable of mediating reactions impossible with single-metal catalysts (Campos, 2020 ▸). Heterometallic drugs that integrate traceability and therapy in one system (theranostic agents) have emerged as a promising alternative to conventional metallodrugs (Redrado *et al.*, 2021 ▸). Theranostic agents are becoming increasingly important in cancer research. Cooperativity of different metals within a single mol­ecular entity is crucial for developing new materials like single-mol­ecule magnets (SMMs), where inter­actions between different types of spin carriers (*e.g*., transition metals and lanthanides) are engineered to achieve high magnetic anisotropy and slow relaxation of magnetization (Shukla *et al.*, 2023 ▸). The combination of distinct metal centres in proximity creates unique electronic structures and enables fine-tuning of light absorption and emission properties (Bonfiglio *et al.*, 2022 ▸). Selective assembly of several different metal ions into a well-defined structure is often synthetically challenging. Overcoming these challenges drives innovation in synthetic methodologies and coordination chemistry.
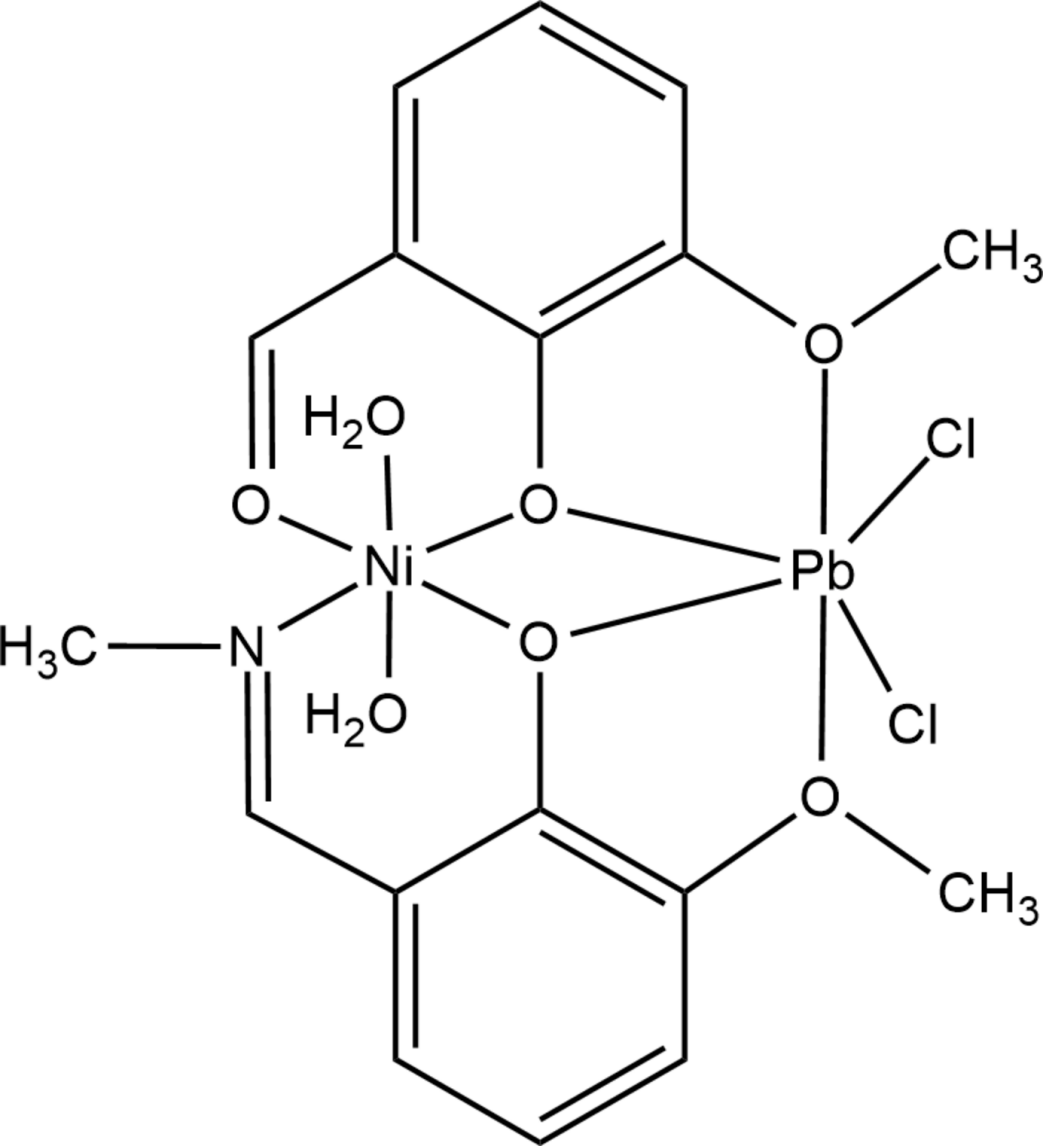


We have recently reported two novel heterometallic mixed-ligand mixed-anion complexes [CuCdCl*L*(*o*-Van)(OAc)]·3H_2_O and [Cu_2_ZnCl_2_*L*_2_(*o*-Van)(OAc)] (GOQHIG and NOTGUB, respectively; Vassilyeva *et al.*, 2025 ▸) synthesized by reacting a fine copper powder and Cd^II^ or Zn^II^ acetate with a methanol solution of the Schiff base ligand H*L* formed *in situ* from 2-hy­droxy-3-meth­oxy-benzaldehyde (*ortho*-vanillin, *o*-VanH) and CH_3_NH_2_·HCl. The Cu/Cd complex demonstrated slow magnetic relaxation under an external magnetic field, a very rarely observable effect in the Cu^II^ coordination compounds.

To continue the series of heterometallics with the 2-meth­oxy-6-[(methyl­imino)­meth­yl]phenol ligand, we report herein on the synthesis and crystal structure of [NiPbCl_2_*L*(*o*-Van)(H_2_O)_2_], (I), prepared using a zerovalent nickel powder and PbCl_2_ as starting materials. Similar to the Cu/Cd and Cu/Zn analogues, the reaction conditions did not favour complete amine-aldehyde condensation, yielding a combination of two kinds of aromatic ligands in complex (I). It is worth noting that the use of two salts in a parallel synthesis did not enable crystallization of a desired hetetometallic product. In the crystal, (I) exhibits full-mol­ecule disorder [occupancy ratio 0.711 (6): 0.289 (6)], generated by a false twofold rotation about the shorter, Ni–Pb, axis of the mol­ecule.

## 2. Structural commentary

**Figure 1:**
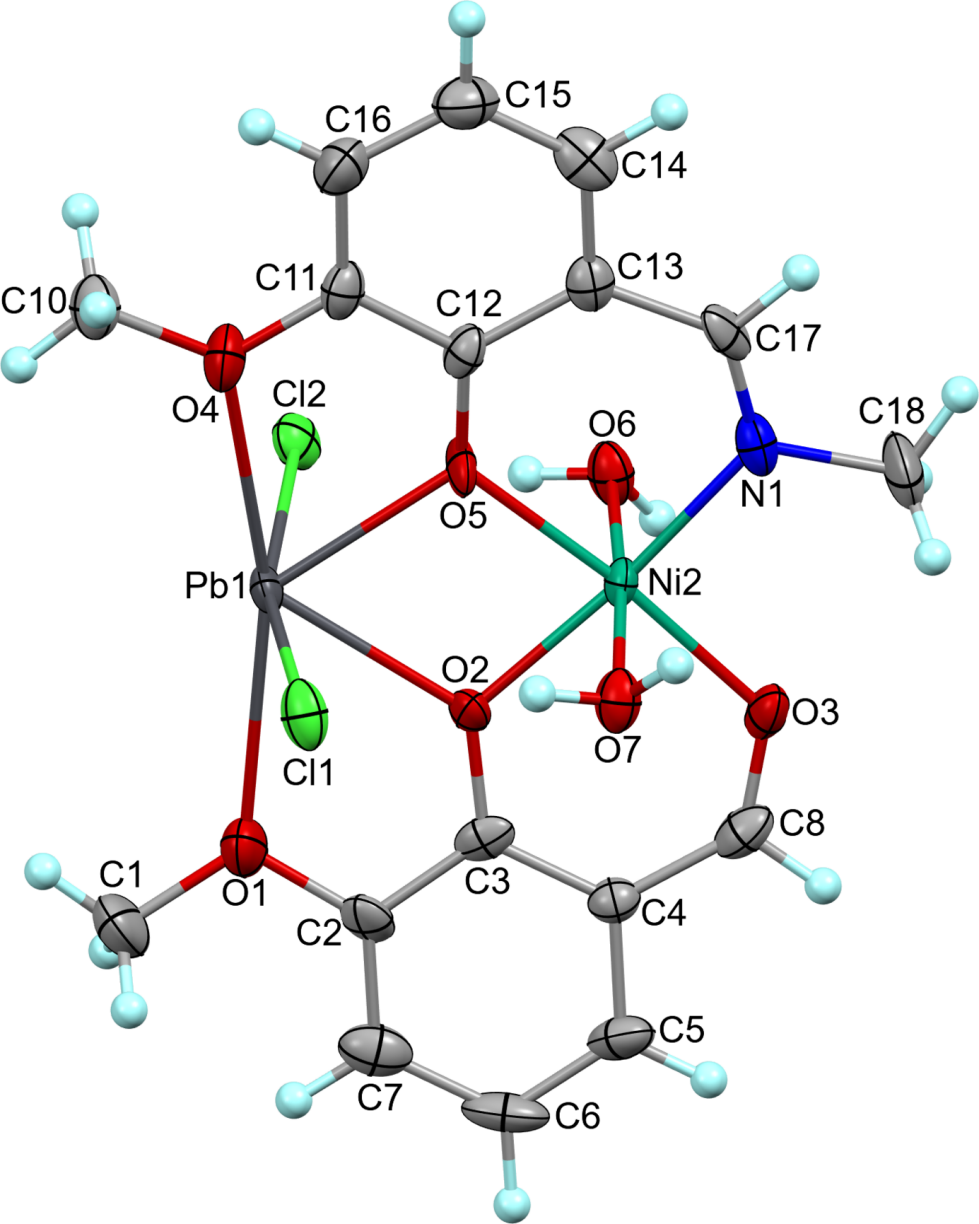
Mol­ecular structure of the major component of the disordered complex [NiPbCl_2_*L*(*o*-Van)(H_2_O)_2_], (I), with atom labelling and displacement ellipsoids drawn at the 50% probability level.

Complex (I) crystallizes in the monoclinic space group *P*2_1_/*c*; the neutral mol­ecule contains two metal centres, the Schiff base and *ortho*-vanillin ligands, both deprotonated, as well as the chloride and aqua ligands. The mol­ecule exhibits a whole-mol­ecule orientational disorder [occupancy ratio 0.711 (6): 0.289 (6)] about a pseudo-twofold rotation axis that roughly bis­ects the mol­ecule along the Ni–Pb axis (Figs. 1 ▸, 2 ▸). The major and minor components slightly differ in bond lengths and angles (Table 1 ▸).

The coordination around the Ni centre is distorted octa­hedral with the four Ni—N/O equatorial bond lengths for the major component falling in the range 1.996 (8)–2.021 (7) Å and the two axial distances to water mol­ecules being slightly longer, 2.088 (6) and 2.090 (5) Å (Table 1 ▸). *Cis* bond angles at the metal atom of the major component vary from 82.3 (2) to 92.6 (6)° and the *trans* angles fall in the range 173.8 (3)–175.4 (5)° (Table 1 ▸). The Pb atom is six-coordinate in a highly distorted tetra­gonal–bipyramidal geometry, the four oxygen atoms from the two ligands are nearly coplanar with the metal centre [Pb—O = 2.301 (8)–2.740 (10) Å] while the two chlorides are located on opposite sides of the plane at Pb—Cl distances of 2.821 (5) and 2.868 (5) Å (major component, Table 1 ▸). The *cis* and *trans* bond angles at the metal atom vary in the ranges 60.8 (3)–129.4 (3) and 132.5 (3)–166.0 (2)°.

The Ni–Pb pair of metals is bridged by two phenolato oxygen atoms, O2/O2*B* and O5/O5*B*, from the two ligands enabling a metal–metal separation of 3.441 (3)/3.477 (7) Å. Most of the mol­ecule, except for the coordinated Cl atoms and water mol­ecules, is nearly planar with the Pb1 atom showing the largest deviation of 0.249 (1) Å from the mean plane defined by the 22 atoms of the major component. The structural configuration of (I) resembles that of GOQHIG, showing similar arrangement of the deprotonated Schiff base and *ortho*-vanillin ligands around the metal centres (Vassilyeva *et al.*, 2025 ▸). The intra­molecular O—H⋯Cl hydrogen bonds involving coordinated H_2_O and chloride ligands appear to be a reason for non-linearity of the axial axes of the Ni and Pb polyhedra (Table 2 ▸).

## 3. Supra­molecular features

**Figure 3:**
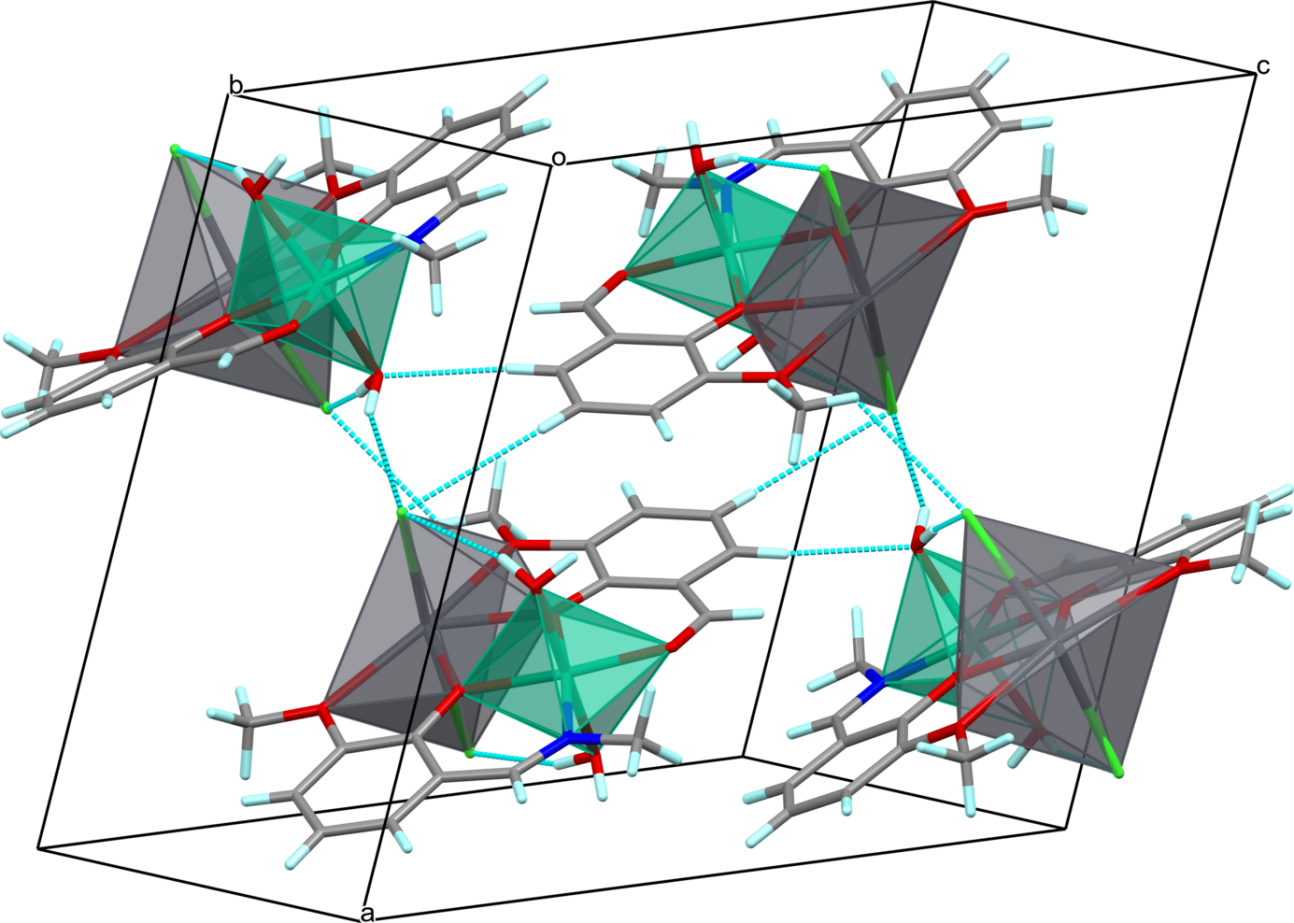
Fragment of the crystal packing of the major component of (I). Green and grey polyhedra denote Ni and Pb atoms, respectively, O atoms are red, N atoms are dark blue, H atoms are light blue, C atoms are grey. Hydrogen bonds are shown as blue dashed lines.

In the solid state, the heterometallic mol­ecules pack loosely (Fig. 3 ▸) and the structure shows no significant inter­molecular contacts; the minimal *M*⋯*M* distance is about 7.65 Å (Ni⋯Pb). The parallel *o*-vanillin rings of the adjacent mol­ecules of (I) display π–π stacking with a ring centroid separation of 3.486 (2) Å (major component). In Fig. 3 ▸, it can be seen that for the major component the mol­ecular packing features O—H⋯Cl and C—H⋯Cl/O hydrogen-bonding inter­actions (Table 2 ▸) that consolidate an extended supra­molecular 3D network structure.

## 4. Database survey

A search in the Cambridge Structural Database for H*L* and its complexes (CSD; Groom *et al.*, 2016 ▸) *via* the WebCSD inter­face in May 2025 revealed 54 original crystal structures, including the structure of the ligand itself. The majority of the homometallic compounds are polynuclear complexes with nuclearity ranging from 2 to 7. Four dimeric (Co, Ni, Cu, Mo), two tetra­meric complexes with cubane- (Mn) or open-cubane type cores (Co), two hexa­metallic Dy compounds with the metal sites adopting a chair-like configuration, and 19 hepta­nuclear hexa­gonal disc-like clusters (Mn, Co, Ni, Zn) have been reported (Meally *et al.*, 2012 ▸). The formation of polymetallic complexes with *L*^−^ of higher nuclearity is usually supported by the presence of other bridging ligands, such as OH^−^, MeO^−^, oxo, acetato or carbamato groups. Mononuclear complexes that possess mol­ecular (Mn, Co, Mo, Cd and Pt) or polymeric structures (Mn, Co) show a higher metal-to-*L*^−^ ratio (1:2 and 1:3). The Schiff base is also able to act as a cation by protonation, counter-balanced by tetra­chloro­cobaltate(II) in [H_2_*L*]_2_CoCl_4_ (KOZQOI; Vassilyeva *et al.*, 2023 ▸).

The heterometallic 1*s*–3*d* examples comprise four structures of Na/*M* (*M* = Fe, Ni) complexes formed in the presence of sodium salts and/or NaOH in the reaction media (Meally *et al.*, 2013 ▸). We have employed the neutral Co*L*_3_ metalloligand to generate a series of heterometallic and mixed-valent [Co^III^*M*^II^*L*_3_Cl_2_]·Solv (*M* = Mn, Co, Zn, Cd; Solv = H_2_O, CH_3_OH) complexes in the absence of other bridging ligands (Nesterova *et al.*, 2018 ▸; Kokozay *et al.*, 2022 ▸). In contrast, the neutral Ni*L*_2_ units required an additional bridging MeO^−^ group to construct the heterometallic dimer [NiZn*L*_2_(OMe)Cl]_2_ (ILIMOI; Vassilyeva *et al.*, 2021 ▸). Similar to (I), the copper-based heterometallics [CuCdCl*L*(*o*-Van)(OAc)]·3H_2_O and [Cu_2_ZnCl_2_*L*_2_(*o*-Van)(OAc)] (Vassilyeva *et al.*, 2025 ▸) use the deprotonated *o*-vanillin mol­ecule to support their integrity.

## 5. Synthesis and crystallization

*o*-Vanillin (0.23 g, 1.5 mmol), CH_3_NH_2_·HCl (0.10 g, 1.5 mmol) and 2-di­methyl­amino­ethanol (0.1 ml, 0.1 mmol) were dissolved in 10 ml of ethanol in a 50 ml conical flask. PbCl_2_ (0.14 g, 0.5 mmol) and Ni powder (0.03 g, 0.5 mmol) were added to the flask under continuous stirring at 333 K. The mixture was stirred magnetically for 2.5 h in the open air until the complete dissolution of the nickel powder and lead salt was observed. The brown solution was filtered and left to evaporate at room temperature. Green plate-like crystals of (I) suitable for X-ray crystallography precipitated the next day. They were filtered off, washed with Pr^*i*^OH and dried in air. An additional amount of the product formed in the mother liquor over several days. Yield: 53%. Analysis calculated for C_17_H_21_Cl_2_NNiO_7_Pb (688.15): C 29.67, H 3.08, N 2.04%. Found: C 29.43, H 2.65, N 1.98%. IR (ν/cm^−1^): 3340br, 3062, 2959, 2922, 2877, 2841, 2792, 1638*s*, 1605, 1553, 1472, 1455*s*, 1441*s*, 1416, 1311, 1290*s*, 1220*s*, 1210*s*, 1106, 1077, 1020, 953, 850, 790, 749, 730, 632, 584, 481, 436.

## 6. Refinement

Crystal data, data collection and structure refinement details are summarized in Table 3 ▸. The heterometallic mol­ecule was modelled as being disordered over two sets of sites with site occupancies refined to 0.711 (6) and its complement. Rigid body restrains (RIGU) were applied to the minor component during refinement. The anisotropic displacement parameters for corresponding atoms in the major and minor components were constrained to be equal. Anisotropic displacement parameters were employed for the non-hydrogen atoms. The water hydrogen atoms were located from the experimental data and refined as rotating groups. Other hydrogen atoms were added at calculated positions and refined as riding with isotropic displacement parameters based on those of the parent atom [C—H = 0.95 Å, *U*_iso_(H) = 1.2*U*_eq_C for CH; C—H = 0.98 Å, *U*_iso_(H) = 1.5*U*_eq_C for CH_3_]. The idealized methyl groups of the major component were refined as rotating groups.

## Supplementary Material

Crystal structure: contains datablock(s) I. DOI: 10.1107/S2056989025005857/ny2013sup1.cif

Structure factors: contains datablock(s) I. DOI: 10.1107/S2056989025005857/ny2013Isup2.hkl

CCDC reference: 2467901

Additional supporting information:  crystallographic information; 3D view; checkCIF report

## Figures and Tables

**Figure 2:**
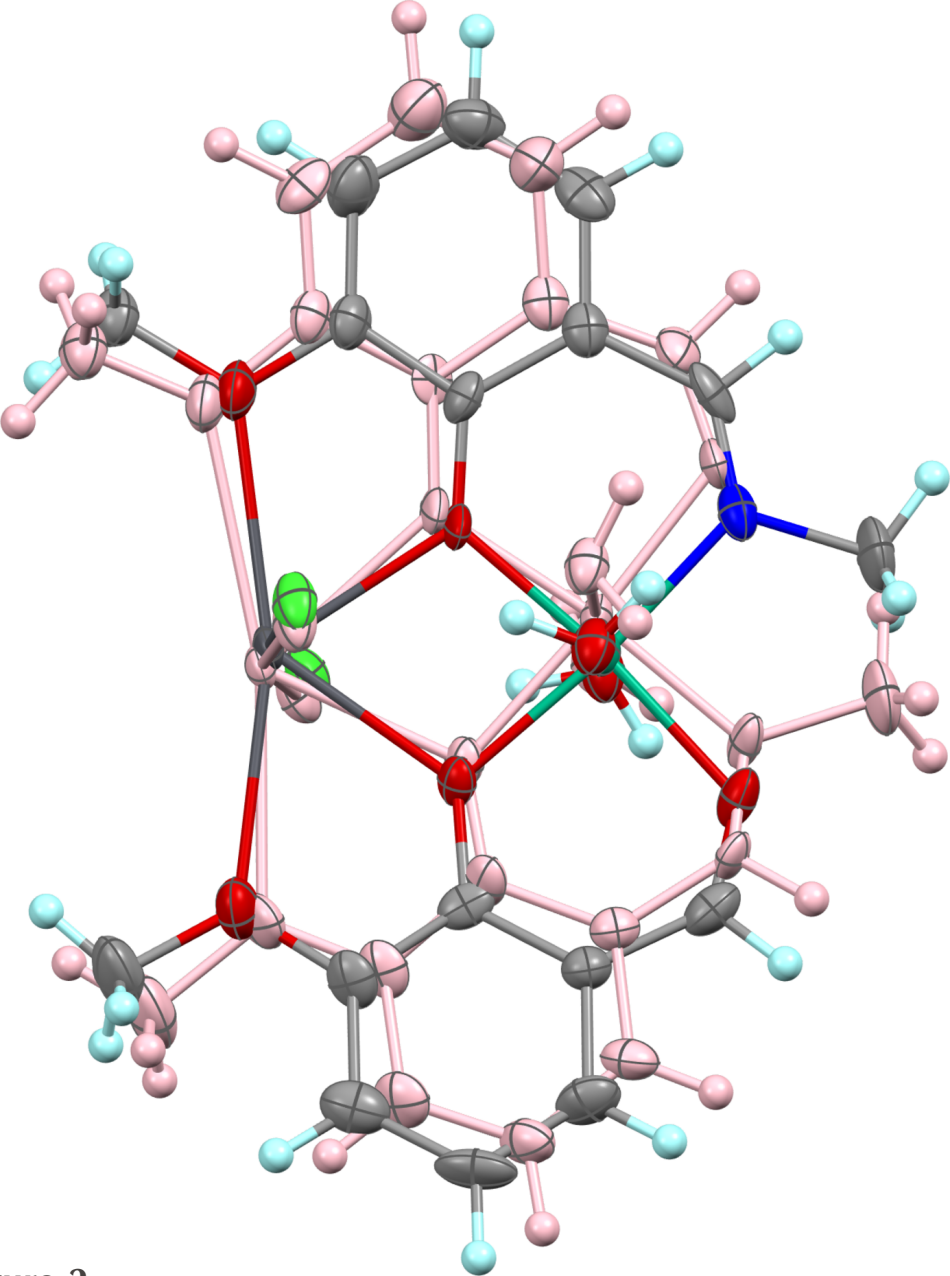
Disposition of the major and minor components of (I) with the minor component shaded in pink.

**Table 1:** Selected geometric parameters (Å, °)

Pb1—Ni2	3.441 (3)	Pb1*B*—Ni2*B*	3.477 (7)
Pb1—Cl1	2.821 (5)	Pb1*B*—Cl1*B*	2.821 (13)
Pb1—Cl2	2.868 (5)	Pb1*B*—Cl2*B*	2.875 (12)
Pb1—O1	2.740 (10)	Pb1*B*—O1*B*	2.762 (19)
Pb1—O2	2.375 (9)	Pb1*B*—O2*B*	2.410 (18)
Pb1—O4	2.663 (9)	Pb1*B*—O4*B*	2.647 (19)
Pb1—O5	2.301 (8)	Pb1*B*—O5*B*	2.302 (18)
Ni2—O2	2.005 (7)	Ni2*B*—O2*B*	1.991 (15)
Ni2—O3	2.021 (7)	Ni2*B*—O3*B*	2.009 (15)
Ni2—O5	2.008 (7)	Ni2*B*—O5*B*	2.016 (16)
Ni2—O6	2.088 (6)	Ni2*B*—O6*B*	2.085 (13)
Ni2—O7	2.090 (5)	Ni2*B*—O7*B*	2.079 (11)
Ni2—N1	1.996 (8)	Ni2*B*—N1*B*	2.003 (14)
			
Cl1—Pb1—Cl2	166.0 (2)	Cl1*B*—Pb1*B*—Cl2*B*	164.7 (5)
O1—Pb1—Cl1	88.5 (4)	O1*B*—Pb1*B*—Cl1*B*	84.9 (11)
O1—Pb1—Cl2	96.9 (4)	O1*B*—Pb1*B*—Cl2*B*	94.7 (9)
O2—Pb1—Cl1	84.8 (4)	O2*B*—Pb1*B*—Cl1*B*	83.2 (9)
O2—Pb1—Cl2	86.7 (3)	O2*B*—Pb1*B*—Cl2*B*	83.5 (8)
O2—Pb1—O1	60.8 (3)	O2*B*—Pb1*B*—O1*B*	59.5 (5)
O2—Pb1—O4	132.5 (3)	O2*B*—Pb1*B*—O4*B*	132.0 (6)
O4—Pb1—Cl1	85.5 (4)	O4*B*—Pb1*B*—Cl1*B*	88.7 (11)
O4—Pb1—Cl2	92.2 (4)	O4*B*—Pb1*B*—Cl2*B*	94.8 (10)
O4—Pb1—O1	164.6 (3)	O4*B*—Pb1*B*—O1*B*	165.9 (7)
O5—Pb1—Cl1	84.2 (3)	O5*B*—Pb1*B*—Cl1*B*	84.5 (9)
O5—Pb1—Cl2	82.4 (3)	O5*B*—Pb1*B*—Cl2*B*	83.6 (8)
O5—Pb1—O1	129.4 (3)	O5*B*—Pb1*B*—O1*B*	126.7 (6)
O5—Pb1—O2	68.7 (2)	O5*B*—Pb1*B*—O2*B*	67.4 (4)
O5—Pb1—O4	64.1 (2)	O5*B*—Pb1*B*—O4*B*	64.8 (6)
O2—Ni2—O3	91.6 (3)	O2*B*—Ni2*B*—O3*B*	94.0 (7)
O2—Ni2—O5	82.3 (2)	O2*B*—Ni2*B*—O5*B*	81.5 (5)
O2—Ni2—O6	92.0 (5)	O2*B*—Ni2*B*—O6*B*	92.7 (13)
O2—Ni2—O7	83.3 (4)	O2*B*—Ni2*B*—O7*B*	83.5 (9)
O3—Ni2—O6	92.6 (6)	O2*B*—Ni2*B*—N1*B*	172.6 (8)
O3—Ni2—O7	87.8 (3)	O3*B*—Ni2*B*—O5*B*	175.3 (8)
O5—Ni2—O3	173.8 (3)	O3*B*—Ni2*B*—O6*B*	92.9 (14)
O5—Ni2—O6	86.7 (6)	O3*B*—Ni2*B*—O7*B*	88.1 (8)
O5—Ni2—O7	92.4 (4)	O5*B*—Ni2*B*—O6*B*	85.8 (14)
O6—Ni2—O7	175.4 (5)	O5*B*—Ni2*B*—O7*B*	92.9 (10)
N1—Ni2—O2	173.9 (4)	O7*B*—Ni2*B*—O6*B*	176.1 (10)
N1—Ni2—O3	94.3 (3)	N1*B*—Ni2*B*—O3*B*	92.8 (7)
N1—Ni2—O5	91.8 (4)	N1*B*—Ni2*B*—O5*B*	91.7 (7)
N1—Ni2—O6	89.1 (6)	N1*B*—Ni2*B*—O6*B*	89.7 (14)
N1—Ni2—O7	95.4 (3)	N1*B*—Ni2*B*—O7*B*	94.0 (9)

**Table 2:** Hydrogen-bond geometry (Å, °)

*D*—H⋯*A*	*D*—H	H⋯*A*	*D*⋯*A*	*D*—H⋯*A*
O6—H2⋯Cl2	0.87	2.27	3.127 (7)	166
O6—H6⋯Cl2^i^	0.87	2.36	3.169 (17)	154
O7—H1⋯Cl1	0.88	2.31	3.152 (8)	162
O7—H7⋯Cl1^ii^	0.88	2.43	3.183 (10)	145
O6*B*—H6*BB*⋯Cl2*B*^i^	0.87	2.14	2.99 (4)	167
C14*B*—H14*B*⋯O6*B*^iii^	0.95	2.50	3.26 (6)	138
C18*B*—H18*D*⋯O3*B*	0.98	2.27	2.96 (3)	127

Symmetry codes: (i) 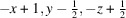; (ii) 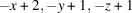; (iii) 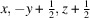.

**Table 3:** Experimental details

Crystal data	
Chemical formula	[NiPb(C_9_H_10_NO_2_)(C_8_H_7_NO_3_)Cl_2_(H_2_O)_2_]
*M*_r_	688.15
Crystal system, space group	Monoclinic, *P*2_1_/*c*
Temperature (K)	150
*a*, *b*, *c* (Å)	14.2171 (6), 9.7678 (3), 16.1291 (6)
β (°)	108.006 (4)
*V* (Å^3^)	2130.15 (14)
*Z*	4
Radiation type	Mo *K*α
μ (mm^−1^)	9.07
Crystal size (mm)	0.44 × 0.15 × 0.06

Data collection	
Diffractometer	New Gemini, Dual, Cu at home/near, Atlas
Absorption correction	Analytical (*CrysAlis PRO*; Rigaku OD, 2023 ▸)
*T*_min_, *T*_max_	0.190, 0.603
No. of measured, independent and observed [*I* > 2σ(*I*)] reflections	13267, 4662, 3785
*R*_int_	0.045
(sin θ/λ)_max_ (Å^−1^)	0.681

Refinement	
*R*[*F*^2^ > 2σ(*F*^2^)], *wR*(*F*^2^), *S*	0.036, 0.082, 1.03
No. of reflections	4662
No. of parameters	437
No. of restraints	461
H-atom treatment	H-atom parameters constrained
Δρ_max_, Δρ_min_ (e Å^−3^)	1.40, −1.55

Computer programs: *CrysAlis PRO* (Rigaku OD, 2023 ▸), *SHELXT2014/4* (Sheldrick, 2015*a* ▸), *SHELXL2016/6* (Sheldrick, 2015*b* ▸), *Mercury* (Macrae *et al.*, 2020 ▸) and *OLEX2* (Dolomanov *et al.*, 2009 ▸).

## supplementary crystallographic information

### Diaqua-1κ2O-dichlorido-2κ2Cl-(µ-2-formyl-6-methoxyphenolato-1κ2O1,O2:2κO6)(µ-2-methoxy-6-[(methylimino)methyl]phenolato-1κ2N,O1:2κO6)lead(II)nickel(II) Crystal data

**Table:**

[NiPb(C_9_H_10_NO_2_)(C_8_H_7_NO_3_)Cl_2_(H_2_O)_2_]	*F*(000) = 1320
*M**_r_* = 688.15	*D*_x_ = 2.146 Mg m^−^^3^
Monoclinic, *P*2_1_/*c*	Mo *K*α radiation, λ = 0.71073 Å
*a* = 14.2171 (6) Å	Cell parameters from 6907 reflections
*b* = 9.7678 (3) Å	θ = 3.1–28.4°
*c* = 16.1291 (6) Å	µ = 9.07 mm^−^^1^
β = 108.006 (4)°	*T* = 150 K
*V* = 2130.15 (14) Å^3^	Plate, green
*Z* = 4	0.44 × 0.15 × 0.06 mm

### Diaqua-1κ2O-dichlorido-2κ2Cl-(µ-2-formyl-6-methoxyphenolato-1κ2O1,O2:2κO6)(µ-2-methoxy-6-[(methylimino)methyl]phenolato-1κ2N,O1:2κO6)lead(II)nickel(II) Data collection

**Table:**

New Gemini, Dual, Cu at home/near, Atlas diffractometer	3785 reflections with *I* > 2σ(*I*)
Detector resolution: 10.6426 pixels mm^-1^	*R*_int_ = 0.045
ω scans	θ_max_ = 29.0°, θ_min_ = 2.5°
Absorption correction: analytical (CrysAlisPro; Rigaku OD, 2023)	*h* = −18→17
*T*_min_ = 0.190, *T*_max_ = 0.603	*k* = −11→12
13267 measured reflections	*l* = −21→19
4662 independent reflections	

### Diaqua-1κ2O-dichlorido-2κ2Cl-(µ-2-formyl-6-methoxyphenolato-1κ2O1,O2:2κO6)(µ-2-methoxy-6-[(methylimino)methyl]phenolato-1κ2N,O1:2κO6)lead(II)nickel(II) Refinement

**Table:**

Refinement on *F*^2^	461 restraints
Least-squares matrix: full	Hydrogen site location: mixed
*R*[*F*^2^ > 2σ(*F*^2^)] = 0.036	H-atom parameters constrained
*wR*(*F*^2^) = 0.082	*w* = 1/[σ^2^(*F*_o_^2^) + (0.0386*P*)^2^ + 2.0045*P*] where *P* = (*F*_o_^2^ + 2*F*_c_^2^)/3
*S* = 1.03	(Δ/σ)_max_ = 0.001
4662 reflections	Δρ_max_ = 1.40 e Å^−^^3^
437 parameters	Δρ_min_ = −1.55 e Å^−^^3^

### Diaqua-1κ2O-dichlorido-2κ2Cl-(µ-2-formyl-6-methoxyphenolato-1κ2O1,O2:2κO6)(µ-2-methoxy-6-[(methylimino)methyl]phenolato-1κ2N,O1:2κO6)lead(II)nickel(II) Special details

**Table:**

Geometry. All esds (except the esd in the dihedral angle between two l.s. planes) are estimated using the full covariance matrix. The cell esds are taken into account individually in the estimation of esds in distances, angles and torsion angles; correlations between esds in cell parameters are only used when they are defined by crystal symmetry. An approximate (isotropic) treatment of cell esds is used for estimating esds involving l.s. planes.

### Diaqua-1κ2O-dichlorido-2κ2Cl-(µ-2-formyl-6-methoxyphenolato-1κ2O1,O2:2κO6)(µ-2-methoxy-6-[(methylimino)methyl]phenolato-1κ2N,O1:2κO6)lead(II)nickel(II) Fractional atomic coordinates and isotropic or equivalent isotropic displacement parameters (Å^2^)

**Table:**

	*x*	*y*	*z*	*U*_iso_*/*U*_eq_	Occ. (<1)
Pb1	0.71331 (18)	0.6780 (2)	0.32661 (18)	0.0192 (2)	0.711 (6)
Ni2	0.75011 (17)	0.3361 (2)	0.37762 (17)	0.0181 (4)	0.711 (6)
Cl1	0.9003 (6)	0.7086 (7)	0.4530 (9)	0.0322 (16)	0.711 (6)
Cl2	0.5382 (4)	0.5785 (8)	0.1987 (5)	0.0273 (12)	0.711 (6)
O1	0.6280 (12)	0.7362 (11)	0.4534 (9)	0.034 (3)	0.711 (6)
O2	0.6904 (9)	0.4990 (8)	0.4181 (6)	0.0210 (13)	0.711 (6)
O3	0.7134 (6)	0.2123 (7)	0.4632 (5)	0.028 (2)	0.711 (6)
O4	0.8114 (10)	0.6915 (7)	0.2104 (9)	0.0322 (16)	0.711 (6)
O5	0.7776 (9)	0.4748 (8)	0.2961 (7)	0.0253 (13)	0.711 (6)
O6	0.6187 (10)	0.2941 (8)	0.2788 (12)	0.0292 (18)	0.711 (6)
H2	0.587543	0.370418	0.260097	0.044*	0.711 (6)
H6	0.579202	0.249284	0.301012	0.044*	0.711 (6)
O7	0.8760 (4)	0.3922 (6)	0.4790 (4)	0.0284 (15)	0.711 (6)
H1	0.895129	0.474209	0.469416	0.043*	0.711 (6)
H7	0.925460	0.339213	0.478427	0.043*	0.711 (6)
N1	0.8164 (7)	0.1881 (8)	0.3310 (6)	0.026 (2)	0.711 (6)
C1	0.6042 (15)	0.8686 (12)	0.4776 (13)	0.055 (5)	0.711 (6)
H1A	0.533645	0.872297	0.472176	0.083*	0.711 (6)
H1B	0.619084	0.937315	0.439202	0.083*	0.711 (6)
H1C	0.643401	0.887320	0.538090	0.083*	0.711 (6)
C2	0.6248 (16)	0.6294 (9)	0.5103 (10)	0.027 (2)	0.711 (6)
C3	0.6581 (16)	0.5046 (13)	0.4869 (11)	0.023 (3)	0.711 (6)
C4	0.6574 (10)	0.3902 (10)	0.5398 (7)	0.0229 (19)	0.711 (6)
C5	0.6239 (10)	0.4051 (14)	0.6130 (7)	0.033 (3)	0.711 (6)
H5	0.624201	0.327941	0.648941	0.040*	0.711 (6)
C6	0.5913 (11)	0.5275 (13)	0.6333 (8)	0.040 (3)	0.711 (6)
H6A	0.569212	0.535901	0.682916	0.048*	0.711 (6)
C7	0.5906 (15)	0.6420 (13)	0.5799 (10)	0.035 (3)	0.711 (6)
H7A	0.566384	0.727548	0.592567	0.042*	0.711 (6)
C8	0.6845 (10)	0.2540 (11)	0.5231 (7)	0.030 (3)	0.711 (6)
H8	0.679333	0.186511	0.563935	0.036*	0.711 (6)
C10	0.8366 (11)	0.8138 (9)	0.1702 (7)	0.032 (2)	0.711 (6)
H10A	0.908445	0.827325	0.190738	0.048*	0.711 (6)
H10B	0.803940	0.893497	0.186018	0.048*	0.711 (6)
H10C	0.814463	0.803010	0.106642	0.048*	0.711 (6)
C11	0.8501 (13)	0.5696 (10)	0.1957 (11)	0.0256 (19)	0.711 (6)
C12	0.8302 (15)	0.4586 (11)	0.2421 (11)	0.022 (3)	0.711 (6)
C13	0.8688 (9)	0.3288 (10)	0.2283 (7)	0.025 (2)	0.711 (6)
C14	0.9206 (9)	0.3185 (11)	0.1681 (8)	0.032 (3)	0.711 (6)
H14	0.944187	0.231385	0.157457	0.039*	0.711 (6)
C15	0.9384 (10)	0.4283 (13)	0.1242 (9)	0.037 (3)	0.711 (6)
H15	0.975374	0.417545	0.084642	0.044*	0.711 (6)
C16	0.9029 (15)	0.5565 (12)	0.1365 (11)	0.038 (3)	0.711 (6)
H16	0.914497	0.633701	0.105225	0.045*	0.711 (6)
C17	0.8558 (9)	0.2032 (11)	0.2714 (7)	0.028 (3)	0.711 (6)
H17	0.879937	0.122232	0.252393	0.034*	0.711 (6)
C18	0.8092 (9)	0.0480 (8)	0.3637 (7)	0.051 (3)	0.711 (6)
H18A	0.739425	0.023298	0.351565	0.077*	0.711 (6)
H18B	0.842460	0.044881	0.426688	0.077*	0.711 (6)
H18C	0.840936	−0.016942	0.334396	0.077*	0.711 (6)
Pb1B	0.7023 (5)	0.6866 (6)	0.3317 (4)	0.0192 (2)	0.289 (6)
Ni2B	0.7554 (5)	0.3385 (6)	0.3531 (4)	0.0181 (4)	0.289 (6)
Cl1B	0.8901 (18)	0.7087 (17)	0.458 (2)	0.0322 (16)	0.289 (6)
Cl2B	0.5250 (12)	0.588 (2)	0.2051 (14)	0.0273 (12)	0.289 (6)
O1B	0.804 (3)	0.724 (2)	0.212 (2)	0.0322 (16)	0.289 (6)
O2B	0.777 (2)	0.4950 (18)	0.2822 (17)	0.0253 (13)	0.289 (6)
O3B	0.8189 (18)	0.2010 (18)	0.2947 (14)	0.032 (5)	0.289 (6)
O4B	0.621 (3)	0.711 (2)	0.458 (2)	0.033 (5)	0.289 (6)
O5B	0.690 (2)	0.4866 (18)	0.4033 (16)	0.0210 (13)	0.289 (6)
O6B	0.614 (2)	0.3004 (19)	0.268 (3)	0.0292 (18)	0.289 (6)
H6BA	0.600144	0.312202	0.211792	0.044*	0.289 (6)
H6BB	0.579774	0.229360	0.273079	0.044*	0.289 (6)
O7B	0.8960 (9)	0.3888 (13)	0.4338 (10)	0.0284 (15)	0.289 (6)
H7BA	0.944470	0.347727	0.416687	0.043*	0.289 (6)
H7BB	0.906628	0.353907	0.488206	0.043*	0.289 (6)
N1B	0.732 (2)	0.1992 (16)	0.4358 (15)	0.026 (4)	0.289 (6)
C1B	0.835 (3)	0.854 (2)	0.193 (2)	0.032 (2)	0.289 (6)
H1BA	0.798451	0.925365	0.213586	0.048*	0.289 (6)
H1BB	0.905674	0.864531	0.222595	0.048*	0.289 (6)
H1BC	0.821185	0.862747	0.130096	0.048*	0.289 (6)
C2B	0.847 (4)	0.609 (2)	0.188 (3)	0.0256 (19)	0.289 (6)
C3B	0.829 (3)	0.488 (2)	0.226 (3)	0.018 (5)	0.289 (6)
C4B	0.869 (3)	0.365 (2)	0.206 (2)	0.025 (2)	0.289 (6)
C5B	0.931 (3)	0.367 (3)	0.152 (3)	0.042 (9)	0.289 (6)
H5B	0.965558	0.286775	0.144337	0.050*	0.289 (6)
C6B	0.940 (4)	0.485 (3)	0.112 (3)	0.043 (7)	0.289 (6)
H6B	0.971557	0.485048	0.067657	0.052*	0.289 (6)
C7B	0.903 (3)	0.609 (3)	0.134 (3)	0.031 (6)	0.289 (6)
H7B	0.917774	0.692505	0.111268	0.038*	0.289 (6)
C8B	0.857 (2)	0.231 (3)	0.2385 (18)	0.027 (6)	0.289 (6)
H8B	0.881052	0.156929	0.213134	0.033*	0.289 (6)
C10B	0.576 (4)	0.833 (3)	0.484 (4)	0.055 (5)	0.289 (6)
H10D	0.578590	0.909605	0.445666	0.083*	0.289 (6)
H10E	0.506607	0.812876	0.478702	0.083*	0.289 (6)
H10F	0.611659	0.856925	0.544445	0.083*	0.289 (6)
C11B	0.625 (4)	0.594 (3)	0.504 (3)	0.027 (2)	0.289 (6)
C12B	0.668 (5)	0.482 (3)	0.477 (3)	0.026 (7)	0.289 (6)
C13B	0.675 (3)	0.358 (2)	0.5260 (19)	0.0229 (19)	0.289 (6)
C14B	0.641 (3)	0.358 (3)	0.598 (2)	0.029 (6)	0.289 (6)
H14B	0.644962	0.275687	0.630598	0.035*	0.289 (6)
C15B	0.603 (3)	0.471 (3)	0.6244 (19)	0.031 (7)	0.289 (6)
H15B	0.582672	0.467078	0.674989	0.037*	0.289 (6)
C16B	0.594 (4)	0.592 (2)	0.579 (2)	0.031 (6)	0.289 (6)
H16B	0.568552	0.671354	0.597384	0.038*	0.289 (6)
C17B	0.708 (3)	0.229 (2)	0.5026 (19)	0.021 (6)	0.289 (6)
H17B	0.711178	0.155661	0.542537	0.025*	0.289 (6)
C18B	0.760 (2)	0.0553 (17)	0.4322 (16)	0.051 (3)	0.289 (6)
H18D	0.778343	0.039607	0.379049	0.077*	0.289 (6)
H18E	0.816853	0.034000	0.483302	0.077*	0.289 (6)
H18F	0.704401	−0.003753	0.431792	0.077*	0.289 (6)

### Diaqua-1κ2O-dichlorido-2κ2Cl-(µ-2-formyl-6-methoxyphenolato-1κ2O1,O2:2κO6)(µ-2-methoxy-6-[(methylimino)methyl]phenolato-1κ2N,O1:2κO6)lead(II)nickel(II) Atomic displacement parameters (Å^2^)

**Table:**

	*U*^11^	*U*^22^	*U*^33^	*U*^12^	*U*^13^	*U*^23^
Pb1	0.0219 (6)	0.0110 (3)	0.0250 (3)	0.0038 (3)	0.0079 (3)	0.0001 (2)
Ni2	0.0202 (5)	0.0119 (3)	0.0219 (12)	−0.0008 (3)	0.0059 (8)	0.0037 (7)
Cl1	0.025 (2)	0.0201 (7)	0.0484 (18)	−0.0036 (9)	0.0074 (19)	−0.0006 (7)
Cl2	0.0245 (18)	0.0245 (13)	0.0312 (14)	0.0086 (13)	0.0061 (15)	0.0001 (10)
O1	0.044 (6)	0.024 (5)	0.033 (5)	0.008 (5)	0.008 (4)	0.004 (4)
O2	0.029 (2)	0.015 (2)	0.018 (4)	−0.0023 (19)	0.004 (3)	−0.003 (2)
O3	0.038 (5)	0.019 (3)	0.024 (7)	−0.008 (3)	0.004 (5)	0.004 (3)
O4	0.034 (3)	0.020 (4)	0.042 (3)	0.004 (4)	0.011 (2)	0.010 (4)
O5	0.030 (2)	0.009 (3)	0.042 (4)	0.000 (2)	0.019 (3)	0.005 (2)
O6	0.026 (2)	0.020 (2)	0.037 (5)	−0.0025 (18)	0.002 (2)	−0.002 (2)
O7	0.024 (3)	0.022 (2)	0.037 (4)	−0.003 (2)	0.006 (3)	0.004 (3)
N1	0.020 (4)	0.016 (4)	0.037 (6)	0.000 (3)	0.003 (4)	−0.002 (4)
C1	0.086 (14)	0.032 (7)	0.046 (5)	0.019 (8)	0.020 (7)	−0.006 (6)
C2	0.031 (3)	0.025 (5)	0.020 (4)	−0.002 (7)	−0.001 (3)	−0.010 (5)
C3	0.016 (7)	0.034 (7)	0.015 (6)	−0.010 (6)	−0.001 (5)	−0.001 (5)
C4	0.021 (6)	0.027 (5)	0.021 (5)	−0.001 (4)	0.007 (3)	0.000 (4)
C5	0.040 (11)	0.044 (10)	0.018 (6)	−0.008 (9)	0.010 (5)	−0.003 (7)
C6	0.032 (6)	0.068 (10)	0.025 (5)	−0.008 (7)	0.015 (4)	−0.020 (6)
C7	0.025 (6)	0.050 (8)	0.029 (5)	−0.002 (8)	0.005 (4)	−0.011 (6)
C8	0.028 (7)	0.036 (6)	0.024 (6)	−0.009 (5)	0.006 (5)	0.007 (5)
C10	0.031 (4)	0.024 (5)	0.039 (7)	−0.004 (5)	0.009 (5)	0.001 (4)
C11	0.023 (3)	0.023 (5)	0.027 (5)	0.003 (6)	0.002 (3)	0.007 (6)
C12	0.023 (6)	0.019 (5)	0.024 (7)	−0.006 (5)	0.004 (6)	0.004 (4)
C13	0.019 (3)	0.023 (5)	0.027 (7)	0.000 (5)	0.000 (4)	0.000 (4)
C14	0.017 (5)	0.036 (6)	0.041 (7)	0.000 (5)	0.005 (4)	−0.009 (5)
C15	0.037 (7)	0.047 (10)	0.028 (8)	0.008 (9)	0.012 (5)	0.001 (8)
C16	0.049 (7)	0.033 (8)	0.029 (6)	0.001 (8)	0.009 (5)	0.007 (7)
C17	0.025 (6)	0.024 (6)	0.027 (8)	0.015 (5)	−0.004 (5)	−0.007 (5)
C18	0.074 (7)	0.014 (4)	0.068 (6)	0.005 (4)	0.027 (5)	−0.001 (4)
Pb1B	0.0219 (6)	0.0110 (3)	0.0250 (3)	0.0038 (3)	0.0079 (3)	0.0001 (2)
Ni2B	0.0202 (5)	0.0119 (3)	0.0219 (12)	−0.0008 (3)	0.0059 (8)	0.0037 (7)
Cl1B	0.025 (2)	0.0201 (7)	0.0484 (18)	−0.0036 (9)	0.0074 (19)	−0.0006 (7)
Cl2B	0.0245 (18)	0.0245 (13)	0.0312 (14)	0.0086 (13)	0.0061 (15)	0.0001 (10)
O1B	0.034 (3)	0.020 (4)	0.042 (3)	0.004 (4)	0.011 (2)	0.010 (4)
O2B	0.030 (2)	0.009 (3)	0.042 (4)	0.000 (2)	0.019 (3)	0.005 (2)
O3B	0.045 (12)	0.013 (7)	0.047 (12)	0.010 (7)	0.027 (10)	0.005 (7)
O4B	0.044 (14)	0.019 (7)	0.041 (12)	−0.002 (8)	0.019 (10)	−0.012 (6)
O5B	0.029 (2)	0.015 (2)	0.018 (4)	−0.0023 (19)	0.004 (3)	−0.003 (2)
O6B	0.026 (2)	0.020 (2)	0.037 (5)	−0.0025 (18)	0.002 (2)	−0.002 (2)
O7B	0.024 (3)	0.022 (2)	0.037 (4)	−0.003 (2)	0.006 (3)	0.004 (3)
N1B	0.051 (14)	0.012 (6)	0.016 (9)	−0.009 (6)	0.010 (7)	−0.003 (6)
C1B	0.031 (4)	0.024 (5)	0.039 (7)	−0.004 (5)	0.009 (5)	0.001 (4)
C2B	0.023 (3)	0.023 (5)	0.027 (5)	0.003 (6)	0.002 (3)	0.007 (6)
C3B	0.013 (11)	0.022 (7)	0.014 (12)	0.007 (10)	−0.006 (8)	0.007 (8)
C4B	0.019 (3)	0.023 (5)	0.027 (7)	0.000 (5)	0.000 (4)	0.000 (4)
C5B	0.052 (18)	0.032 (15)	0.05 (2)	0.000 (15)	0.028 (15)	0.003 (13)
C6B	0.058 (19)	0.038 (15)	0.036 (17)	−0.009 (14)	0.017 (13)	0.000 (13)
C7B	0.026 (12)	0.033 (14)	0.033 (12)	−0.013 (14)	0.006 (9)	0.002 (13)
C8B	0.022 (12)	0.024 (8)	0.038 (15)	0.007 (9)	0.012 (12)	0.004 (9)
C10B	0.086 (14)	0.032 (7)	0.046 (5)	0.019 (8)	0.020 (7)	−0.006 (6)
C11B	0.031 (3)	0.025 (5)	0.020 (4)	−0.002 (7)	−0.001 (3)	−0.010 (5)
C12B	0.04 (2)	0.017 (7)	0.018 (10)	−0.004 (9)	0.008 (11)	−0.007 (7)
C13B	0.021 (6)	0.027 (5)	0.021 (5)	−0.001 (4)	0.007 (3)	0.000 (4)
C14B	0.025 (14)	0.036 (13)	0.030 (11)	−0.003 (13)	0.015 (9)	−0.007 (11)
C15B	0.037 (18)	0.029 (14)	0.033 (14)	−0.008 (14)	0.019 (11)	−0.011 (12)
C16B	0.037 (16)	0.029 (13)	0.026 (10)	−0.007 (14)	0.007 (9)	−0.014 (11)
C17B	0.032 (16)	0.017 (8)	0.014 (13)	−0.003 (9)	0.006 (12)	0.006 (8)
C18B	0.074 (7)	0.014 (4)	0.068 (6)	0.005 (4)	0.027 (5)	−0.001 (4)

### Diaqua-1κ2O-dichlorido-2κ2Cl-(µ-2-formyl-6-methoxyphenolato-1κ2O1,O2:2κO6)(µ-2-methoxy-6-[(methylimino)methyl]phenolato-1κ2N,O1:2κO6)lead(II)nickel(II) Geometric parameters (Å, º)

**Table:**

Pb1—Ni2	3.441 (3)	Pb1B—Cl1B	2.821 (13)
Pb1—Cl1	2.821 (5)	Pb1B—Cl2B	2.875 (12)
Pb1—Cl2	2.868 (5)	Pb1B—O1B	2.762 (19)
Pb1—O1	2.740 (10)	Pb1B—O2B	2.410 (18)
Pb1—O2	2.375 (9)	Pb1B—O4B	2.647 (19)
Pb1—O4	2.663 (9)	Pb1B—O5B	2.302 (18)
Pb1—O5	2.301 (8)	Ni2B—O2B	1.991 (15)
Ni2—O2	2.005 (7)	Ni2B—O3B	2.009 (15)
Ni2—O3	2.021 (7)	Ni2B—O5B	2.016 (16)
Ni2—O5	2.008 (7)	Ni2B—O6B	2.085 (13)
Ni2—O6	2.088 (6)	Ni2B—O7B	2.079 (11)
Ni2—O7	2.090 (5)	Ni2B—N1B	2.003 (14)
Ni2—N1	1.996 (8)	Cl1B—O7B	3.152 (15)
Cl1—O7	3.152 (8)	Cl1B—O7B^i^	3.15 (2)
Cl2—O6	3.127 (7)	Cl2B—O6B	3.123 (15)
O1—C1	1.422 (14)	Cl2B—O6B^ii^	2.99 (4)
O1—C2	1.399 (13)	O1B—C1B	1.41 (2)
O2—C3	1.327 (12)	O1B—C2B	1.39 (2)
O3—C8	1.231 (12)	O2B—C3B	1.33 (2)
O4—C10	1.457 (12)	O3B—C8B	1.224 (19)
O4—C11	1.363 (11)	O4B—C10B	1.47 (2)
O5—C12	1.323 (12)	O4B—C11B	1.36 (2)
O6—H2	0.8719	O5B—C12B	1.32 (2)
O6—H6	0.8718	O6B—H6BA	0.8700
O7—H1	0.8752	O6B—H6BB	0.8700
O7—H7	0.8751	O7B—H7BA	0.9111
N1—C17	1.262 (14)	O7B—H7BB	0.9090
N1—C18	1.481 (11)	N1B—C17B	1.26 (2)
C1—H1A	0.9800	N1B—C18B	1.468 (18)
C1—H1B	0.9800	C1B—H1BA	0.9800
C1—H1C	0.9800	C1B—H1BB	0.9800
C2—C3	1.401 (14)	C1B—H1BC	0.9800
C2—C7	1.361 (15)	C2B—C3B	1.39 (2)
C3—C4	1.407 (13)	C2B—C7B	1.35 (2)
C4—C5	1.410 (13)	C3B—C4B	1.41 (2)
C4—C8	1.434 (13)	C4B—C5B	1.42 (2)
C5—H5	0.9500	C4B—C8B	1.438 (19)
C5—C6	1.358 (15)	C5B—H5B	0.9500
C6—H6A	0.9500	C5B—C6B	1.35 (2)
C6—C7	1.408 (16)	C6B—H6B	0.9500
C7—H7A	0.9500	C6B—C7B	1.41 (2)
C8—H8	0.9500	C7B—H7B	0.9500
C10—H10A	0.9800	C8B—H8B	0.9500
C10—H10B	0.9800	C10B—H10D	0.9800
C10—H10C	0.9800	C10B—H10E	0.9800
C11—C12	1.395 (12)	C10B—H10F	0.9800
C11—C16	1.390 (15)	C11B—C12B	1.39 (2)
C12—C13	1.425 (13)	C11B—C16B	1.39 (2)
C13—C14	1.392 (14)	C12B—C13B	1.43 (2)
C13—C17	1.450 (14)	C13B—C14B	1.39 (2)
C14—H14	0.9500	C13B—C17B	1.44 (2)
C14—C15	1.350 (15)	C14B—H14B	0.9500
C15—H15	0.9500	C14B—C15B	1.35 (2)
C15—C16	1.387 (15)	C15B—H15B	0.9500
C16—H16	0.9500	C15B—C16B	1.37 (2)
C17—H17	0.9500	C16B—H16B	0.9500
C18—H18A	0.9800	C17B—H17B	0.9500
C18—H18B	0.9800	C18B—H18D	0.9800
C18—H18C	0.9800	C18B—H18E	0.9800
Pb1B—Ni2B	3.477 (7)	C18B—H18F	0.9800
			
Cl1—Pb1—Ni2	83.70 (15)	O1B—Pb1B—Cl2B	94.7 (9)
Cl1—Pb1—Cl2	166.0 (2)	O2B—Pb1B—Ni2B	33.8 (4)
Cl2—Pb1—Ni2	82.93 (15)	O2B—Pb1B—Cl1B	83.2 (9)
O1—Pb1—Ni2	95.3 (2)	O2B—Pb1B—Cl2B	83.5 (8)
O1—Pb1—Cl1	88.5 (4)	O2B—Pb1B—O1B	59.5 (5)
O1—Pb1—Cl2	96.9 (4)	O2B—Pb1B—O4B	132.0 (6)
O2—Pb1—Ni2	34.57 (18)	O4B—Pb1B—Ni2B	98.4 (5)
O2—Pb1—Cl1	84.8 (4)	O4B—Pb1B—Cl1B	88.7 (11)
O2—Pb1—Cl2	86.7 (3)	O4B—Pb1B—Cl2B	94.8 (10)
O2—Pb1—O1	60.8 (3)	O4B—Pb1B—O1B	165.9 (7)
O2—Pb1—O4	132.5 (3)	O5B—Pb1B—Ni2B	33.7 (4)
O4—Pb1—Ni2	98.17 (16)	O5B—Pb1B—Cl1B	84.5 (9)
O4—Pb1—Cl1	85.5 (4)	O5B—Pb1B—Cl2B	83.6 (8)
O4—Pb1—Cl2	92.2 (4)	O5B—Pb1B—O1B	126.7 (6)
O4—Pb1—O1	164.6 (3)	O5B—Pb1B—O2B	67.4 (4)
O5—Pb1—Ni2	34.18 (18)	O5B—Pb1B—O4B	64.8 (6)
O5—Pb1—Cl1	84.2 (3)	O2B—Ni2B—Pb1B	42.3 (5)
O5—Pb1—Cl2	82.4 (3)	O2B—Ni2B—O3B	94.0 (7)
O5—Pb1—O1	129.4 (3)	O2B—Ni2B—O5B	81.5 (5)
O5—Pb1—O2	68.7 (2)	O2B—Ni2B—O6B	92.7 (13)
O5—Pb1—O4	64.1 (2)	O2B—Ni2B—O7B	83.5 (9)
O2—Ni2—Pb1	42.2 (3)	O2B—Ni2B—N1B	172.6 (8)
O2—Ni2—O3	91.6 (3)	O3B—Ni2B—Pb1B	136.3 (5)
O2—Ni2—O5	82.3 (2)	O3B—Ni2B—O5B	175.3 (8)
O2—Ni2—O6	92.0 (5)	O3B—Ni2B—O6B	92.9 (14)
O2—Ni2—O7	83.3 (4)	O3B—Ni2B—O7B	88.1 (8)
O3—Ni2—Pb1	133.8 (2)	O5B—Ni2B—Pb1B	39.3 (5)
O3—Ni2—O6	92.6 (6)	O5B—Ni2B—O6B	85.8 (14)
O3—Ni2—O7	87.8 (3)	O5B—Ni2B—O7B	92.9 (10)
O5—Ni2—Pb1	40.1 (3)	O6B—Ni2B—Pb1B	88.5 (6)
O5—Ni2—O3	173.8 (3)	O7B—Ni2B—Pb1B	88.2 (4)
O5—Ni2—O6	86.7 (6)	O7B—Ni2B—O6B	176.1 (10)
O5—Ni2—O7	92.4 (4)	N1B—Ni2B—Pb1B	130.9 (5)
O6—Ni2—Pb1	88.5 (2)	N1B—Ni2B—O3B	92.8 (7)
O6—Ni2—O7	175.4 (5)	N1B—Ni2B—O5B	91.7 (7)
O7—Ni2—Pb1	87.88 (16)	N1B—Ni2B—O6B	89.7 (14)
N1—Ni2—Pb1	131.9 (3)	N1B—Ni2B—O7B	94.0 (9)
N1—Ni2—O2	173.9 (4)	Pb1B—Cl1B—O7B	83.7 (4)
N1—Ni2—O3	94.3 (3)	Pb1B—Cl1B—O7B^i^	156.0 (10)
N1—Ni2—O5	91.8 (4)	O7B^i^—Cl1B—O7B	73.2 (6)
N1—Ni2—O6	89.1 (6)	Pb1B—Cl2B—O6B	83.7 (4)
N1—Ni2—O7	95.4 (3)	O6B^ii^—Cl2B—O6B	143.0 (9)
Pb1—Cl1—O7	82.82 (18)	C1B—O1B—Pb1B	122.7 (17)
Pb1—Cl2—O6	83.03 (18)	C2B—O1B—Pb1B	117.1 (13)
C1—O1—Pb1	126.3 (8)	C2B—O1B—C1B	118 (2)
C2—O1—Pb1	116.5 (7)	Ni2B—O2B—Pb1B	103.9 (8)
C2—O1—C1	116.0 (10)	C3B—O2B—Pb1B	131.4 (13)
Ni2—O2—Pb1	103.2 (3)	C3B—O2B—Ni2B	124.5 (14)
C3—O2—Pb1	129.7 (7)	C8B—O3B—Ni2B	123.3 (16)
C3—O2—Ni2	126.6 (7)	C10B—O4B—Pb1B	128.6 (18)
C8—O3—Ni2	123.9 (6)	C11B—O4B—Pb1B	113.2 (13)
C10—O4—Pb1	127.5 (6)	C11B—O4B—C10B	118 (2)
C11—O4—Pb1	114.0 (6)	Ni2B—O5B—Pb1B	107.1 (8)
C11—O4—C10	118.1 (9)	C12B—O5B—Pb1B	123.8 (15)
Ni2—O5—Pb1	105.8 (4)	C12B—O5B—Ni2B	127.1 (15)
C12—O5—Pb1	126.0 (6)	Ni2B—O6B—Cl2B	105.4 (6)
C12—O5—Ni2	127.9 (6)	Ni2B—O6B—H6BA	122.0
Ni2—O6—Cl2	105.5 (3)	Ni2B—O6B—H6BB	122.4
Ni2—O6—H2	109.6	Cl2B^iii^—O6B—Cl2B	116.7 (12)
Ni2—O6—H6	108.8	Cl2B—O6B—H6BA	66.6
Cl2—O6—H2	10.2	Cl2B^iii^—O6B—H6BA	106.9
Cl2—O6—H6	114.6	Cl2B—O6B—H6BB	124.3
H2—O6—H6	104.4	Cl2B^iii^—O6B—H6BB	9.1
Ni2—O7—Cl1	104.8 (2)	H6BA—O6B—H6BB	104.5
Ni2—O7—H1	109.8	Ni2B—O7B—Cl1B	104.7 (5)
Ni2—O7—H7	109.5	Ni2B—O7B—H7BA	112.1
Cl1—O7—H1	13.1	Ni2B—O7B—H7BB	110.9
Cl1—O7—H7	117.2	Cl1B^i^—O7B—Cl1B	106.8 (6)
H1—O7—H7	104.2	Cl1B^i^—O7B—H7BA	49.0
C17—N1—Ni2	125.1 (7)	Cl1B—O7B—H7BA	121.7
C17—N1—C18	118.4 (9)	Cl1B—O7B—H7BB	104.8
C18—N1—Ni2	116.3 (7)	Cl1B^i^—O7B—H7BB	60.5
O1—C1—H1A	109.5	H7BA—O7B—H7BB	102.2
O1—C1—H1B	109.5	C17B—N1B—Ni2B	124.1 (16)
O1—C1—H1C	109.5	C17B—N1B—C18B	113.7 (18)
H1A—C1—H1B	109.5	C18B—N1B—Ni2B	121.4 (13)
H1A—C1—H1C	109.5	O1B—C1B—H1BA	109.5
H1B—C1—H1C	109.5	O1B—C1B—H1BB	109.5
O1—C2—C3	113.0 (10)	O1B—C1B—H1BC	109.5
C7—C2—O1	124.5 (10)	H1BA—C1B—H1BB	109.5
C7—C2—C3	122.4 (10)	H1BA—C1B—H1BC	109.5
O2—C3—C2	119.6 (10)	H1BB—C1B—H1BC	109.5
O2—C3—C4	122.9 (10)	O1B—C2B—C3B	114.4 (18)
C2—C3—C4	117.5 (9)	C7B—C2B—O1B	125.7 (18)
C3—C4—C5	119.5 (9)	C7B—C2B—C3B	119.9 (18)
C3—C4—C8	124.8 (9)	O2B—C3B—C2B	117.5 (18)
C5—C4—C8	115.6 (9)	O2B—C3B—C4B	123.5 (18)
C4—C5—H5	119.3	C2B—C3B—C4B	118.9 (17)
C6—C5—C4	121.4 (9)	C3B—C4B—C5B	120.4 (17)
C6—C5—H5	119.3	C3B—C4B—C8B	125.7 (18)
C5—C6—H6A	120.3	C5B—C4B—C8B	113.9 (18)
C5—C6—C7	119.4 (10)	C4B—C5B—H5B	120.8
C7—C6—H6A	120.3	C6B—C5B—C4B	118 (2)
C2—C7—C6	119.7 (11)	C6B—C5B—H5B	120.8
C2—C7—H7A	120.1	C5B—C6B—H6B	119.7
C6—C7—H7A	120.1	C5B—C6B—C7B	121 (2)
O3—C8—C4	129.0 (9)	C7B—C6B—H6B	119.7
O3—C8—H8	115.5	C2B—C7B—C6B	121 (2)
C4—C8—H8	115.5	C2B—C7B—H7B	119.5
O4—C10—H10A	109.5	C6B—C7B—H7B	119.5
O4—C10—H10B	109.5	O3B—C8B—C4B	128 (2)
O4—C10—H10C	109.5	O3B—C8B—H8B	115.8
H10A—C10—H10B	109.5	C4B—C8B—H8B	115.8
H10A—C10—H10C	109.5	O4B—C10B—H10D	109.5
H10B—C10—H10C	109.5	O4B—C10B—H10E	109.5
O4—C11—C12	115.2 (10)	O4B—C10B—H10F	109.5
O4—C11—C16	122.2 (9)	H10D—C10B—H10E	109.5
C16—C11—C12	122.6 (9)	H10D—C10B—H10F	109.5
O5—C12—C11	120.7 (9)	H10E—C10B—H10F	109.5
O5—C12—C13	122.2 (8)	O4B—C11B—C12B	115.8 (19)
C11—C12—C13	117.1 (9)	O4B—C11B—C16B	120.9 (18)
C12—C13—C17	124.4 (9)	C12B—C11B—C16B	123.0 (19)
C14—C13—C12	119.0 (8)	O5B—C12B—C11B	121 (2)
C14—C13—C17	116.6 (9)	O5B—C12B—C13B	122.2 (18)
C13—C14—H14	118.9	C11B—C12B—C13B	116.7 (17)
C15—C14—C13	122.3 (9)	C12B—C13B—C17B	124.8 (18)
C15—C14—H14	118.9	C14B—C13B—C12B	119.0 (17)
C14—C15—H15	119.8	C14B—C13B—C17B	116.1 (18)
C14—C15—C16	120.4 (10)	C13B—C14B—H14B	119.0
C16—C15—H15	119.8	C15B—C14B—C13B	122 (2)
C11—C16—H16	120.7	C15B—C14B—H14B	119.0
C15—C16—C11	118.6 (10)	C14B—C15B—H15B	119.5
C15—C16—H16	120.7	C14B—C15B—C16B	121 (2)
N1—C17—C13	128.1 (9)	C16B—C15B—H15B	119.5
N1—C17—H17	116.0	C11B—C16B—H16B	120.9
C13—C17—H17	116.0	C15B—C16B—C11B	118 (2)
N1—C18—H18A	109.5	C15B—C16B—H16B	120.9
N1—C18—H18B	109.5	N1B—C17B—C13B	128 (2)
N1—C18—H18C	109.5	N1B—C17B—H17B	115.9
H18A—C18—H18B	109.5	C13B—C17B—H17B	115.9
H18A—C18—H18C	109.5	N1B—C18B—H18D	109.5
H18B—C18—H18C	109.5	N1B—C18B—H18E	109.5
Cl1B—Pb1B—Ni2B	82.8 (4)	N1B—C18B—H18F	109.5
Cl1B—Pb1B—Cl2B	164.7 (5)	H18D—C18B—H18E	109.5
Cl2B—Pb1B—Ni2B	81.9 (4)	H18D—C18B—H18F	109.5
O1B—Pb1B—Ni2B	93.2 (4)	H18E—C18B—H18F	109.5
O1B—Pb1B—Cl1B	84.9 (11)		
			
Pb1—O1—C2—C3	−6 (2)	Pb1B—O1B—C2B—C3B	2 (6)
Pb1—O1—C2—C7	176.0 (17)	Pb1B—O1B—C2B—C7B	−177 (4)
Pb1—O2—C3—C2	5 (3)	Pb1B—O2B—C3B—C2B	1 (6)
Pb1—O2—C3—C4	−173.7 (13)	Pb1B—O2B—C3B—C4B	179 (3)
Pb1—O4—C11—C12	1 (2)	Pb1B—O4B—C11B—C12B	−1 (7)
Pb1—O4—C11—C16	179.4 (15)	Pb1B—O4B—C11B—C16B	173 (4)
Pb1—O5—C12—C11	−1 (3)	Pb1B—O5B—C12B—C11B	17 (9)
Pb1—O5—C12—C13	178.8 (12)	Pb1B—O5B—C12B—C13B	−171 (4)
Ni2—O2—C3—C2	175.3 (15)	Ni2B—O2B—C3B—C2B	−173 (3)
Ni2—O2—C3—C4	−3 (3)	Ni2B—O2B—C3B—C4B	4 (6)
Ni2—O3—C8—C4	9.3 (18)	Ni2B—O3B—C8B—C4B	4 (5)
Ni2—O5—C12—C11	−173.8 (13)	Ni2B—O5B—C12B—C11B	179 (5)
Ni2—O5—C12—C13	6 (3)	Ni2B—O5B—C12B—C13B	−9 (9)
Ni2—N1—C17—C13	4.9 (16)	Ni2B—N1B—C17B—C13B	12 (7)
O1—C2—C3—O2	2 (3)	O1B—C2B—C3B—O2B	−2 (7)
O1—C2—C3—C4	−179.7 (18)	O1B—C2B—C3B—C4B	180 (5)
O1—C2—C7—C6	−179.7 (18)	O1B—C2B—C7B—C6B	−178 (5)
O2—C3—C4—C5	178.6 (17)	O2B—C3B—C4B—C5B	−174 (4)
O2—C3—C4—C8	−4 (3)	O2B—C3B—C4B—C8B	3 (7)
O4—C11—C12—O5	0 (3)	O4B—C11B—C12B—O5B	−10 (10)
O4—C11—C12—C13	179.7 (17)	O4B—C11B—C12B—C13B	178 (5)
O4—C11—C16—C15	−178.9 (17)	O4B—C11B—C16B—C15B	−177 (5)
O5—C12—C13—C14	177.7 (16)	O5B—C12B—C13B—C14B	−174 (5)
O5—C12—C13—C17	−1 (3)	O5B—C12B—C13B—C17B	1 (9)
C1—O1—C2—C3	−174.3 (19)	C1B—O1B—C2B—C3B	166 (4)
C1—O1—C2—C7	8 (3)	C1B—O1B—C2B—C7B	−13 (8)
C2—C3—C4—C5	0 (3)	C2B—C3B—C4B—C5B	3 (7)
C2—C3—C4—C8	176.9 (17)	C2B—C3B—C4B—C8B	−179 (4)
C3—C2—C7—C6	2 (3)	C3B—C2B—C7B—C6B	3 (8)
C3—C4—C5—C6	1 (2)	C3B—C4B—C5B—C6B	−8 (7)
C3—C4—C8—O3	1 (2)	C3B—C4B—C8B—O3B	−8 (7)
C4—C5—C6—C7	0 (2)	C4B—C5B—C6B—C7B	11 (8)
C5—C4—C8—O3	178.1 (13)	C5B—C4B—C8B—O3B	170 (4)
C5—C6—C7—C2	−2 (2)	C5B—C6B—C7B—C2B	−9 (8)
C7—C2—C3—O2	180 (2)	C7B—C2B—C3B—O2B	177 (5)
C7—C2—C3—C4	−2 (3)	C7B—C2B—C3B—C4B	−1 (8)
C8—C4—C5—C6	−176.6 (12)	C8B—C4B—C5B—C6B	174 (4)
C10—O4—C11—C12	174.6 (15)	C10B—O4B—C11B—C12B	180 (6)
C10—O4—C11—C16	−7 (3)	C10B—O4B—C11B—C16B	−6 (8)
C11—C12—C13—C14	−2 (2)	C11B—C12B—C13B—C14B	−2 (9)
C11—C12—C13—C17	179.6 (14)	C11B—C12B—C13B—C17B	173 (5)
C12—C11—C16—C15	−1 (3)	C12B—C11B—C16B—C15B	−4 (9)
C12—C13—C14—C15	2 (2)	C12B—C13B—C14B—C15B	−1 (8)
C12—C13—C17—N1	−6 (2)	C12B—C13B—C17B—N1B	−3 (8)
C13—C14—C15—C16	−2 (2)	C13B—C14B—C15B—C16B	1 (8)
C14—C13—C17—N1	176.1 (12)	C14B—C13B—C17B—N1B	173 (5)
C14—C15—C16—C11	1 (3)	C14B—C15B—C16B—C11B	1 (8)
C16—C11—C12—O5	−178 (2)	C16B—C11B—C12B—O5B	176 (6)
C16—C11—C12—C13	2 (3)	C16B—C11B—C12B—C13B	4 (10)
C17—C13—C14—C15	−179.4 (12)	C17B—C13B—C14B—C15B	−176 (4)
C18—N1—C17—C13	178.5 (11)	C18B—N1B—C17B—C13B	−179 (4)

Symmetry codes: (i) −*x*+2, −*y*+1, −*z*+1; (ii) −*x*+1, *y*+1/2, −*z*+1/2; (iii) −*x*+1, *y*−1/2, −*z*+1/2.

### Diaqua-1κ2O-dichlorido-2κ2Cl-(µ-2-formyl-6-methoxyphenolato-1κ2O1,O2:2κO6)(µ-2-methoxy-6-[(methylimino)methyl]phenolato-1κ2N,O1:2κO6)lead(II)nickel(II) Hydrogen-bond geometry (Å, º)

**Table:**

*D*—H···*A*	*D*—H	H···*A*	*D*···*A*	*D*—H···*A*
O6—H2···Cl2	0.87	2.27	3.127 (7)	166
O6—H6···Cl2^iii^	0.87	2.36	3.169 (17)	154
O7—H1···Cl1	0.88	2.31	3.152 (8)	162
O7—H7···Cl1^i^	0.88	2.43	3.183 (10)	145
O6*B*—H6*BB*···Cl2*B*^iii^	0.87	2.14	2.99 (4)	167
C14*B*—H14*B*···O6*B*^iv^	0.95	2.50	3.26 (6)	138
C18*B*—H18*D*···O3*B*	0.98	2.27	2.96 (3)	127

Symmetry codes: (i) −*x*+2, −*y*+1, −*z*+1; (iii) −*x*+1, *y*−1/2, −*z*+1/2; (iv) *x*, −*y*+1/2, *z*+1/2.
